# Supplementary material for: Inter/Intra-Observer Agreement in Video-Capsule Endoscopy: Are We Getting It All Wrong? A Systematic Review and Meta-Analysis
Source: Diagnostics (Basel). 2022 Oct 2;12(10):2400. doi: 10.3390/diagnostics12102400 (PMC9600122; doi:10.3390/diagnostics12102400)
Supplement: Supplementary file 1 [file diagnostics-12-02400-s001.zip › diagnostics-1947766-supplementary.pdf]

Supplementary Material

Table S1: search strings with PICO questions, Search string:

Investigation	Outcome
Capsule camera*	Interobserv*
Wireless camera*	Intraobserv*
Wireless camera endoscop*	Inter-observ*
WCE	Intra-observ*
CCE	Inter observ*
Colon capsule endoscop*	Intra observ*
PillCam*	Interrater reliability/
Pill Cam*	Observer variation [Mesh]
Camera pill*	Observer agreement*
Capsule endoscopy/	Observer disagreement*
Capsule endoscope/	Observer Variations
Capsule endoscop*	Variation, Observer
Endoscop*, Capsule	Variations, Observer
Wireless Capsule Endoscop*	Bias, Observer
Capsule Endoscop*, Wireless	Observer Bias
Endoscop*, Wireless Capsule	Interobserver Variation
Video Capsule Endoscop*	Interobserver Variations
Capsule Endoscop*, Video	Variation, Interobserver
Capsule Endoscopy, Video	Variations, Interobserver
Endoscop*, Video Capsule	Inter-Observer Variation
Pan-enteric capsule endoscop*	Inter Observer Variation
Capsule endoscopy [MeSH]	Inter-Observer Variations
	Variation, Inter-Observer
	Variations, Inter-Observer
	Interobserver Variability
	Interobserver Variabilities
	Variabilities, Interobserver
	Variability, Interobserver
	Inter-Observer Variability
	Inter Observer Variability
	Inter-Observer Variabilities
	Variabilities, Inter-Observer
	Variability, Inter-Observer
	Intraobserver Variation
	Intraobserver Variations
	Variation, Intraobserver
	Variations, Intraobserver
	Intra-Observer Variation
	Intra Observer Variation
	Intra-Observer Variations
	Variation, Intra-Observer
	Variations, Intra-Observer
	Intraobserver Variability
	Intraobserver Variabilities
	Variabilities, Intraobserver
	Variability, Intraobserver
	Intra-Observer Variability

	Intra Observer Variability<br>Intra-Observer Variabilities<br>Variabilities, Intra-Observer<br>Variability, Intra-Observer<br>Inter-observer agreement<br>Inter-observer concordance<br>Inter-observer reliability<br>Inter-rater agreement<br>Inter-rater concordance<br>Inter-rater reliability<br>Interobserver agreement<br>Interobserver concordance<br>Interobserver reliability<br>Interrater agreement<br>Interrater concordance<br>Interrater reliability

* represents any group of characters including no character. / =subject heading Embase. MeSH = mesh term pubmed.

Question: What are the overall inter- and intra-observer agreements and variation in colon capsule endoscopy (CCE) reading?

P: Not applicable

I: colon capsule endoscopy

C: Not applicable

O: inter/intra-observer agreement
